# The dysfunction of complement and coagulation in diseases: the implications for the therapeutic interventions

**DOI:** 10.1002/mco2.785

**Published:** 2024-10-23

**Authors:** Honghong Jiang, Yiming Guo, Qihang Wang, Yiran Wang, Dingchuan Peng, Yigong Fang, Lei Yan, Zhuolin Ruan, Sheng Zhang, Yong Zhao, Wendan Zhang, Wei Shang, Zhichun Feng

**Affiliations:** ^1^ Faculty of Pediatrics, the Seventh Medical Center of Chinese PLA General Hospital National Engineering Laboratory for Birth Defects Prevention and Control of Key Technology, Beijing Key Laboratory of Pediatric Organ Failure Beijing China; ^2^ Department of Biological Science, The Dietrich School of Arts and Sciences University of Pittsburgh Pittsburgh Pennsylvania USA; ^3^ Department of Obstetrics and Gynecology The Seventh Medical Center of Chinese PLA General Hospital Beijing China; ^4^ Department of Obstetrics and Gynecology The sixth Medical Center of Chinese PLA General Hospital Beijing China; ^5^ School of Medicine South China University of Technology Guangzhou China; ^6^ Institute of Acupuncture and Moxibustion China Academy of Chinese Medical Sciences Beijing China; ^7^ Department of Obstetrics and Gynecology，Chinese PLA General Hospital Beijing China

**Keywords:** coagulation, complement system, immune response, inflammatory network, sepsis

## Abstract

The complement system, comprising over 30 proteins, is integral to the immune system, and the coagulation system is critical for vascular homeostasis. The activation of the complement and coagulation systems involves an organized proteolytic cascade, and the overactivation of these systems is a central pathogenic mechanism in several diseases. This review describes the role of complement and coagulation system activation in critical illness, particularly sepsis. The complexities of sepsis reveal significant knowledge gaps that can be compared to a profound abyss, highlighting the urgent need for further investigation and exploration. It is well recognized that the inflammatory network, coagulation, and complement systems are integral mechanisms through which multiple factors contribute to increased susceptibility to infection and may result in a disordered immune response during septic events in patients. Given the overlapping pathogenic mechanisms in sepsis, immunomodulatory therapies currently under development may be particularly beneficial for patients with sepsis who have concurrent infections. Herein, we present recent findings regarding the molecular relationships between the coagulation and complement pathways in the advancement of sepsis, and propose potential intervention targets related to the crosstalk between coagulation and complement, aiming to provide more valuable treatment of sepsis.

## Graphical abstract

Understanding the alterations in complement and coagulation systems could aid in the creation of novel therapeutic approaches to tackle the dysfunction of complement and coagulation in diseases. The unacceptably high mortality rate of sepsis has prompted further research into the molecular pathogenesis of sepsis and therapeutic interventions. The present review aims to treat diseases by targeting complement and coagulation pathways

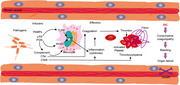

## 1 INTRODUCTION

**FIGURE 1:**
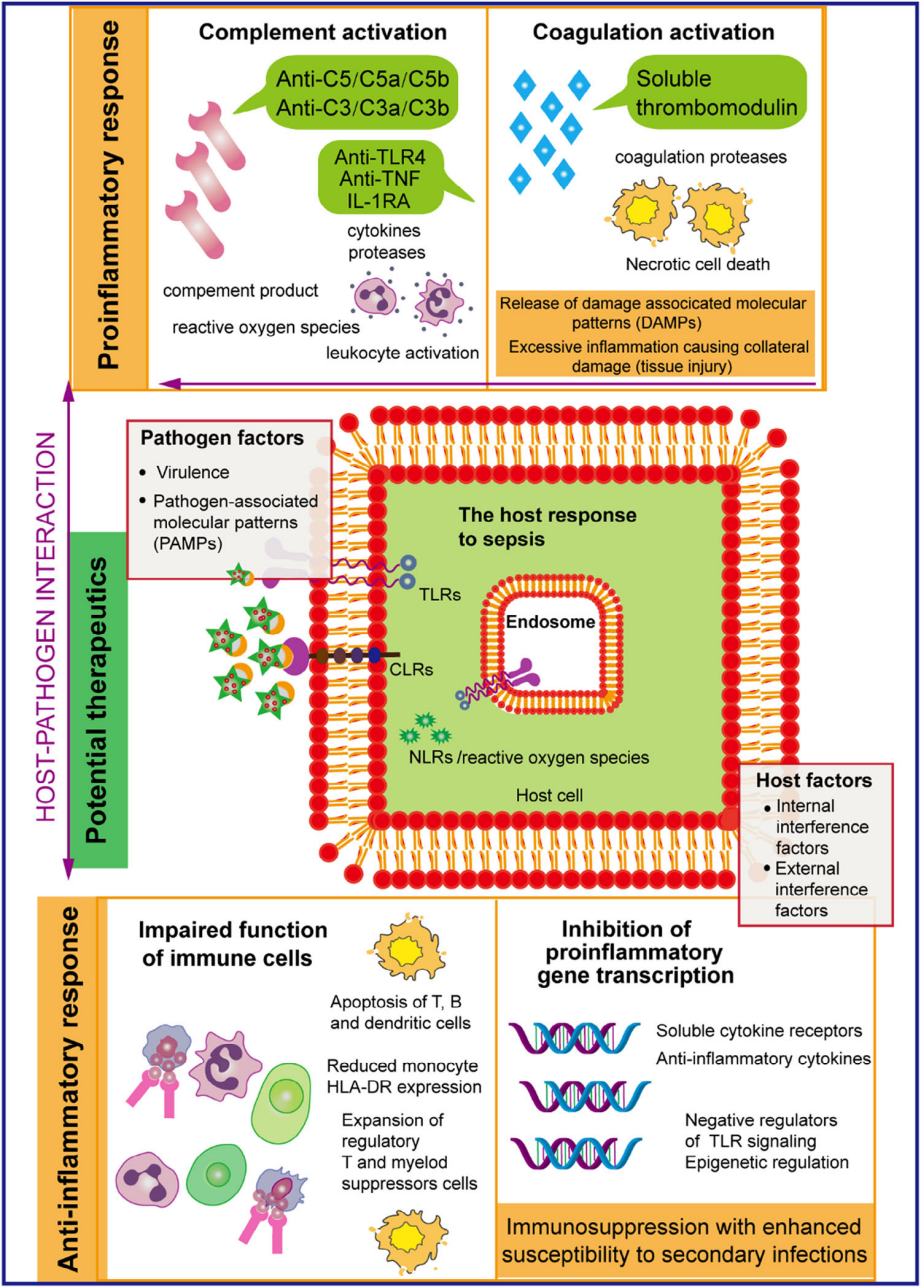
The immunopathogenesis of sepsis is a complex process that begins when the immune system detects an invading pathogen. This initial recognition is facilitated by various receptors located on the cell surface and within the cells themselves, known as pattern recognition receptors. These receptors are specifically designed to identify pathogen‐associated molecular patterns (PAMPs), triggering the body's inflammatory response. The effectiveness and scope of the immune response can vary significantly depending on several factors, including the type of pathogen involved and the individual characteristics of the host. Unfortunately, when the host response becomes dysregulated, it can lead to severe outcomes such as organ failure and, ultimately, death. The proinflammatory aspect of the immune response involves a cascade of events marked by the release of proinflammatory mediators from various cell types throughout the body. This phase includes the activation of systems crucial for maintaining homeostasis, such as the complement and coagulation systems, as well as the vascular endothelium. Consequently, this response can also lead to the release of alarmins or damage‐associated molecular patterns (DAMPs), which can further intensify the proinflammatory response, causing additional tissue damage. Therefore, the interplay between inflammation and tissue injury is a critical element in the progression of sepsis. Conversely, the anti‐inflammatory response presents a different set of challenges. It is characterized by a decline in the functionality of immune cells and a reduced ability to produce proinflammatory cytokines. This impaired response is influenced by a variety of factors, including epigenetic mechanisms that alter gene expression, the secretion of anti‐inflammatory cytokines, and the presence of negative regulators that affect toll‐like receptor (TLR) signaling. Understanding these intricate dynamics is essential for developing effective immunomodulatory interventions in the treatment of sepsis. IL, interleukin; TNF, tumor necrosis factor.

The complement system, which was first identified in the 1890s, consists of over 30 soluble proteins and proteins anchored to cell membranes.^1^, ^2^ Initially, the prevailing view was that the primary function of the complement system was to support innate immunity.^3^ However, emerging research has revealed that this complex system also plays a critical role in adaptive immune responses, particularly those involving T cells and B cells.^4^, ^5^ In addition to its established functions in both innate and adaptive immunity, recent findings indicate that the complement system is also implicated in various physiological processes, including tissue regeneration and tumor growth, highlighting its multifaceted nature in both health and disease. For instance, it contributes to the formation and refinement of neuronal synapses, which are essential for proper brain function and communication between nerve cells.^6^ Additionally, complement plays a significant role in promoting tissue regeneration and repair, highlighting its importance in maintaining overall tissue health and facilitating recovery from injury. This multifunctional nature of the complement system underscores its importance not only in immunity but also in various physiological processes essential for the growth and healing of tissues.^5^, ^7^, ^8^ However, its dysregulation or overactivation can lead to tissue damage, such as the systemic inflammatory response, including thrombotic inflammation due to hemostasis, observed in trauma and sepsis, as well as severe COVID‐19.^9^, ^10^ Damage‐associated molecular patterns (DAMPs) generated during ischemia–reperfusion injuries (myocardial infarction, stroke, and transplant dysfunction) and in chronic neuropathy and rheumatism activate complement, thereby increasing destructive inflammation^11^, ^12^, ^13^ (Figure 1). Recent studies have provided growing evidence indicating that the complement system may act as a key pathological factor in several prevalent diseases. This emerging understanding opens up new possibilities for the development of targeted complement therapies.^14^ By exploring these avenues, researchers could potentially create innovative treatment options aimed at alleviating or managing conditions where complement dysregulation plays a significant role.^15^ Such advancements could ultimately enhance patient outcomes and offer new hope for those affected by these common illnesses.^16^, ^17^

The coagulation‐associated protease system plays a crucial role in the body's ability to maintain vascular homeostasis, which is essential for overall health and function. This system is intricately regulated and must maintain a delicate balance in order to respond effectively to various pathophysiological stimuli.^18^ Such stimuli can range from injury and infection to other conditions that may disrupt normal blood flow and vascular integrity. Proper functioning of this protease system ensures that the body can manage and adjust its coagulation processes as needed, safeguarding against both excessive bleeding and unwanted clot formation.^19^, ^20^, ^21^ The coagulation system is essential component in the development and progression of various major diseases.^22^ Its roles are particularly prominent in the context of pathological processes where inflammation is a key feature. A notable example of this phenomenon is observed in cases of sepsis, a life‐threatening condition characterized by a systemic inflammatory response.^23^ During such episodes, there is a marked and significant activation of both the complement and coagulation systems, which underscores their interconnectedness and importance in the inflammatory response associated with this severe condition.^24^ In sepsis, excessive inflammatory storm activation of the immune response accompanied by the release of a series of proinflammatory cytokines, including tumor necrosis factor (TNF), interleukin (IL)‐6, and IL‐1β, and so forth^25^, ^26^, ^27^ further deteriorating into activation of the NETosis and complement systems, which can have deleterious effects on host tissues through the development of microcirculatory disorders, which in turn causes the activation of the coagulation system,^20^ including the adhesion and aggregation of platelets through on von Willebrand factor (VWF) and coagulation factors,^26^, ^28^ leading to the formation of microthrombi, which subsequently leads to disseminated intravascular coagulation (DIC), further exacerbating the process.^29^, ^30^ The various cascade reactions of the complement system are activated and play crucial roles in numerous biological processes. Notably, the protease components involved in coagulation, along with their associated regulatory factors, are instrumental in modulating the hemostatic‐thrombotic response.^31^, ^32^ However, their influence extends beyond this singular function. These components are also key participants in a variety of essential biological processes, which encompass both innate and adaptive immunity, the regulation of inflammation, the promotion of cell proliferation, the mechanisms of wound healing, and even the complexities of cancer development. This multifaceted involvement highlights their significance in maintaining overall physiological balance and responding to diverse pathological conditions.^22^, ^33^, ^34^ To sum up, the disorder of the coagulation system may have a profound and malformed effect on the homeostasis of the body (Figure 1).

Sepsis is a critical public health problem that imposes an unbearable economic burden around the world, severely reducing quality of life and longevity.^15^, ^35^, ^36^, ^37^ The pathophysiology of sepsis and septic shock is remarkably intricate, as it encompasses a wide array of physiological pathways that interact with one another.^38^ Initially, the process begins with the invasion of pathogen‐associated molecular patterns (PAMPs) or DAMPs into the host organism.^18^, ^39^ This invasion leads to the production of endotoxins or similar harmful substances that enter the bloodstream, which in turn triggers an inflammatory response characterized by the release of various mediators intended to act locally to protect the organism from infection.^40^ However, the sustained release of these mediators sets into motion a complex series of regulatory mechanisms that involve both the upregulation and downregulation of numerous immune pathways within the system.^15^ This dysregulation ultimately results in significant metabolic abnormalities and can precipitate organ dysfunction.^41^ The interplay of these immune pathways highlights the delicate balance the body must maintain to effectively address infection while avoiding the detrimental consequences of an excessive immune response.^42^ Understanding these dynamics is essential for creating more effective interventions to manage and mitigate the severe impacts of sepsis.^43^, ^44^ The introduction of these factors into the bloodstream triggers three primary cascade pathways: the complement system, the inflammatory response, and the coagulation and fibrinolytic pathways. Each of these pathways plays a crucial role in the body's response to infection and injury.^45^ It has been demonstrated that genetic polymorphisms in various components of these pathways are linked to an individual's susceptibility to sepsis, the severity of the condition, and the overall outcome of infectious shock.^46^ Understanding these associations is vital for developing tailored approaches to treatment and management of sepsis and related conditions.^47^ The unacceptably high mortality rate has prompted further research into the molecular pathogenesis of sepsis and improved therapeutic interventions.^39^, ^48^ Insights into the pathogenic mechanisms underlying sepsis and septic shock progression are emerging and highlight the critical role of immune hyperreactivity, characterized by extensive endothelial damage, complement‐induced coagulation, and systemic microangiopathy, in disease exacerbation.

The symbiotic relationship between the coagulation and complement systems is pivotal in the vicissitudes of sepsis, with modulation of these mechanisms potentially mitigating the organ impairment and mortality rates affiliated with the condition.^49^ Profound insights into the activation and interplay of these systems are imperative for unraveling the enigmatic pathophysiological tapestry of sepsis.^42^ The coagulation and complement cascades emerge from a common phylogenetic lineage, their intricate symbiosis is paramount for the host's innate defense mechanisms against invading pathogens.^15^ However, within the context of sepsis, this equilibrium is susceptible to perturbations, precipitating a cascade of hyperinflammation and unbridled coagulation.^18^ Complement components possess the agency to incite the conversion of zymogens within the coagulation cascade to their active protease forms, and vice versa.^50^ Illustratively, the anaphylatoxin C5a and the terminal complement complex (C5b‐9) are capable of inducing endothelial expression of tissue factor (TF), a pivotal initiator of the extrinsic coagulation pathway.^51^, ^52^, ^53^ Conversely, the catalytic action of TF alongside coagulation factors FVIIa, FXa, thrombin, and fibrin can stimulate G protein‐coupled protease‐activated receptors, thereby instigating a proinflammatory signaling cascade.^54^ The elucidation of these interwoven mechanisms, which are subject to a dynamic and complex modulation during the sepsis pathophysiological process, is fundamental for the advancement of efficacious therapeutic interventions.

Inquiry into the coagulation and complement systems traverses a spectrum of academic domains, encompassing immunology, hematology, critical care medicine, and molecular biology, thereby fostering a confluence of interdisciplinary efforts and the amalgamation of intellectual capital.^55^ Scrutinizing the modulatory functions of the coagulation and complement systems within the sepsis paradigm is indispensable for augmenting our cognizance of this lethal affliction, optimizing patient stewardship, and innovating therapeutic modalities.^3^ A compendium of potential therapeutic targets is instrumental in alleviating the strain on medical infrastructure and bolstering the tenets of public health.^46^ Furthermore, the aberrant activation of the coagulation and complement systems is discernible in the nascent phases of sepsis.^49^, ^55^ Delving into their nuanced roles across a spectrum of patient profiles not only catalyzes the advent of personalized therapeutics, crafting efficacious treatment regimens for heterogenous patient collectives, but also engenders the genesis of novel biomarkers.^56^ These biomarkers act as a benchmark for the appraisal of extant pharmacological interventions and the inception of innovative medicinal entities, thus facilitating the preemptive identification and prompt intervention of sepsis. Here, we aim to provide detailed insights into how complement and coagulation contribute to host clearance of inflammation and restore homeostasis during the development of sepsis. In particular, we will focus on the recognition mechanisms that drive the activation of these cascades against inflammatory immunity.

## 2 CROSSTALK OF COAGULATION AND COMPLEMENT IN SEPSIS

Sepsis is characterized by a systemic inflammatory response and concomitant organ dysfunction. The complement and coagulation systems play pivotal roles in the pathogenesis of sepsis, influencing each other to form a complex interactive network. The role of the complement system in sepsis is primarily manifested through the generation of complement fragments, such as C3a and C5a, upon its activation. These fragments possess potent proinflammatory activity, capable of promoting the recruitment and activation of leukocytes, endothelial cells, and platelets. However, unregulated complement activation may lead to tissue damage and result in organ failure. The coagulation system's role in sepsis is evidenced by the development of DIC, a clinical syndrome characterized by the activation of both the coagulation and fibrinolytic systems within the vasculature. In sepsis, the coagulation system's activation becomes dysregulated, predisposing the microvasculature to thrombosis, which, if severe, can lead to multiorgan failure. In summary, the complement and coagulation systems exert a critical influence on the pathogenesis of sepsis. Their activation can be considered part of the host's initial immune response to infection, yet excessive activation may precipitate pathological harm. Therefore, modulating the activation of these systems, especially in the treatment of sepsis, represents a significant therapeutic strategy.

### 2.1 Activation of complement in sepsis

The complement system is the humoral part of the innate immune system consisting of approximately 30 serum proteins.^57^, ^58^, ^59^ The system consists of a serine protease cascade reaction, which involves a series of sequential cleavages of complement proteins. This intricate process ultimately leads to the formation of the membrane attack complex (MAC).^13^, ^50^ The MAC plays a crucial role by creating a pore in the cellular membrane of the target cell where the complement system has been activated. Moreover, the complement system that circulates within the body can be activated through at least three distinct pathways.^32^ Of these pathways, the classical, lectin, and alternative pathways are the most well established and widely recognized. Each of these pathways contributes to the overall function of the complement system in immune responses, facilitating the elimination of pathogens and damaged cells.^60^, ^61^ The classical pathway of the complement system is initiated when C1q attaches to complexes formed between antigens and antibodies. This binding event marks the beginning of a complex series of biochemical reactions that ultimately help to eliminate pathogens.^27^ In contrast, the lectin pathway is activated when mannose‐binding lectins (MBLs) or ficolins interact with specific sugars located on the surface of bacterial cell walls. Both pathways work through enzymatic processes that lead to a selective cleavage of complement proteins C3 and C5, performed by enzymes that are referred to as C3 convertases and C5 convertases, respectively. This limited cleavage plays a crucial role in amplifying the immune response and facilitating the destruction of invading microorganisms.^62^, ^63^ The alternative pathway of complement activation involves the spontaneous hydrolysis of C3, leading to its convergence with two other pathways at the C3 convertase enzyme. This enzyme plays a crucial role in cleaving C3, resulting in the production of the proinflammatory peptide known as C3a, alongside a significant quantity of C3b.^70^ The generation of C3b is particularly important as it acts to condition the pathogen, enhancing the immune response. Furthermore, C3b also plays a vital role in the formation of the C5 convertase, an important complex that catalyzes the release of the potent allergenic toxin C5a. This fragment, C5a, is instrumental in eliciting a robust immune response. In addition to C5a, the process also produces the fragment C5b, which is critical for assembling the MAC known as C5b‐9.^23^, ^65^ This complex forms on the surface of target cells, marking the culmination of the complement activation process.^66^, ^67^ The release of allergenic toxins, such as C3a and C5a, during complement activation is associated with significant proinflammatory effects.^60^, ^68^ These include an increase in vascular permeability and the recruitment of leukocytes, both of which contribute to the body's defensive response. However, it is important to note that when complement activation occurs in an uncontrolled manner, it can lead to an overwhelming inflammatory response, resulting in substantial damage to host tissues.

Moreover, an additional mechanism contributing to the production of C5 activation products involves the ligation of leukocyte proteases.^69^ The result of this process is the generation of two allergenic toxins, namely, C3a and C5a, which induce distinct alterations in the acute inflammatory response while also leading to smooth muscle contraction. C5a, in particular, is recognized as a potent agonist for myeloid cells, notably neutrophils.^19^, ^70^ These cells are characterized by the expression of high levels of C5a receptors (C5aRs), also referred to as C5aR or CD88. Neutrophils not only respond to various chemical stimuli but also exhibit a range of additional biological reactions that are crucial for the immune response.^4^, ^71^, ^72^ Nonmyeloid cells, including fine bronchial and alveolar epithelial cells, as well as endothelial cells, have been found to express the C5aR and to have responses to the chemotactic factor C5a.^74^, ^81^ This indicates that these nonmyeloid cell types are not merely passive participants in immune responses but actively engage with complement components, highlighting their roles in initiating or modulating inflammatory signals.^75^, ^76^, ^77^ Furthermore, C3a has the capacity to interact with a diverse range of receptors across various cell types.^77^, ^78^ One of the well‐documented effects of C3a is its ability to induce deplasticization of basophils and mast cells, which results in the release of vasoactive amines. This release is significant as it contributes to the processes of edema and contraction of smooth muscle, particularly affecting the upper respiratory tract and the intestinal system, which are crucial for maintaining homeostasis during immune responses.^40^ In addition, C3b exhibits a distinct mechanism by binding to the surfaces of bacteria, thereby endowing these pathogens with an “exogenous” characteristic. This modification facilitates their rapid recognition and uptake by phagocytic cells, which possess specific receptors for C3b and its related products.^8^ The binding of C3b not only enhances the efficiency of phagocytosis but also underscores the importance of complement activation in the innate immune response, as it enables the immune system to effectively clear invading microorganisms from the host.

### 2.2 Coagulation activation of sepsis

DIC is a life‐threatening pathophysiological syndrome characterized by increases the systemic overactivation of blood coagulation, leading to microvascular thrombosis and multiple organ dysfunctions, such as sepsis.^56^, ^79^ During sepsis, there is a rapid increase in the production of pro‐ and anti‐inflammatory cytokines in the blood stream, and their abnormal production will contribute to massive activation of coagulation factors and platelets as well as damage to the vascular endothelium, which result in vascular leakage and DIC.^14^, ^80^ In addition to ensuring that thrombus production after activation of the coagulation system, advanced DIC may also cause bleeding when platelets and coagulation factors are depleted.^22^, ^81^ These conditions often lead to extensive crosstalks between inflammation and coagulation, which can eventually lead to multiple organ dysfunction syndrome (MODS) and eventual death.

In the early stages of sepsis, proinflammatory cytokines are crucial in influencing the hemostatic system. They exert their effects through several mechanisms.^82^, ^83^ First, these cytokines can induce dysfunction in endothelial cells, which are vital for maintaining vascular health and proper blood flow. Second, there may be an increase in platelet activation, leading to a heightened risk of abnormal clot formation. Additionally, the activation of coagulation may be mediated by TFs, which are substances that initiate the clotting process in response to vascular injury.^50^, ^65^, ^66^ Furthermore, the functioning of physiological anticoagulation pathways is often impaired during this phase, disrupting the natural balance that prevents excessive clotting. Finally, the overall fibrinolytic system, which is responsible for breaking down clots, can also be perturbed, further complicating the hemostatic response. Together, these pathways reflect the complex interplay of inflammatory processes and hemostatic changes that occur in sepsis.^7^, ^84^ In this period, changes in coagulation profile are dynamic, and once the initial symptoms improve, the function of global coagulation homeostasis would be restored.^85^, ^86^ Previous studies have suggestion that the hemostatic balance in the organism is always shifted toward the activation of coagulation, either by activation of the coagulation pathway or by downregulation of the anticoagulation pathway.^87^, ^88^ The interaction between the intrinsic immune system and the hemostatic system, which includes platelets and coagulation factors, plays a critical role in the development of sepsis. This collaboration is significant because it highlights how the body's defensive mechanisms and the blood clotting process are interconnected.^89^, ^90^ By understanding this relationship, researchers can gain insights into the complex pathophysiology of sepsis, potentially leading to more effective interventions and treatments for this serious condition.^91^, ^92^ In sepsis patients, activation of coagulation coincides with the release of inflammation‐associated mediators, which also is characteristic of systemic inflammatory response syndromes.^93^, ^94^, ^95^

### 2.3 Coagulation and inflammatory network

The coagulation system is traditionally considered to be an integral part of homeostasis maintenance and a completely independent system that function is to prevent or limit the loss of blood volume and blood components after mechanical injury to the circulatory system, in addition to being the main promoter of thrombosis (pathological formation of intravascular thrombi).^68^, ^96^ Research has confirmed that the interplay between inflammation and coagulation is deemed pivotal to the pathogenesis of sepsis. Inflammation can induce a coagulation response, and in turn, an activated coagulation response can further promote the occurrence of inflammatory reactions.^40^ During the onset of sepsis, there is a disruption characterized by impaired protein synthesis, continuous consumption of three pathways of coagulation inhibitors, and a relatively low level of protein degradation.^93^, ^97^ In the context of sepsis, inflammatory mediators such as TNF‐α, IL‐1, and IL‐6 are implicated in the activation of coagulation and the suppression of fibrinolysis by influencing the activation of the coagulation and fibrinolytic systems.^98^ These inflammatory mediators not only facilitate the coagulation response but also, upon activation of coagulation, they can further stimulate the release of inflammatory mediators, establishing a crosstalk between inflammation and coagulation.^99^, ^100^ Furthermore, during sepsis, the activation of coagulation predominantly occurs via the TF pathway. TF, primarily derived from monocytes or vascular endothelial cells, activates factor VII in the circulation, which in turn activates factor X, culminating in the conversion of prothrombin to thrombin.^19^ Conversely, the anticoagulant pathways in sepsis, encompassing antithrombin, the protein C system, and tissue factor pathway inhibitor (TFPI), are severely compromised, leading to a reduction in antithrombin activity and an impairment of the functional capacity of activated protein C.

Moreover, the fibrinolytic system is in a state of inhibition during sepsis, with the fibrinolytic process being dampened at two critical junctures: the swift neutralization of plasmin by alpha‐2 antiplasmin and the inactivation of plasminogen activators (PAs) by plasminogen activator inhibitor‐1 (PAI‐1).^101^ Collectively, these elements culminate in the excessive production of fibrin and the formation of microvascular thrombi, which in turn precipitate organ dysfunction.^87^ In aggregate, the intricate interplay of the coagulation system within the sepsis paradigm encompasses the liberation of inflammatory mediators, the ignition of coagulative cascades, the undermining of anticoagulant mechanisms, and the subjugation of the fibrinolytic apparatus.^102^ These interrelated elements synergize to advance the sepsis trajectory and engender organ injury.

**FIGURE 2:**
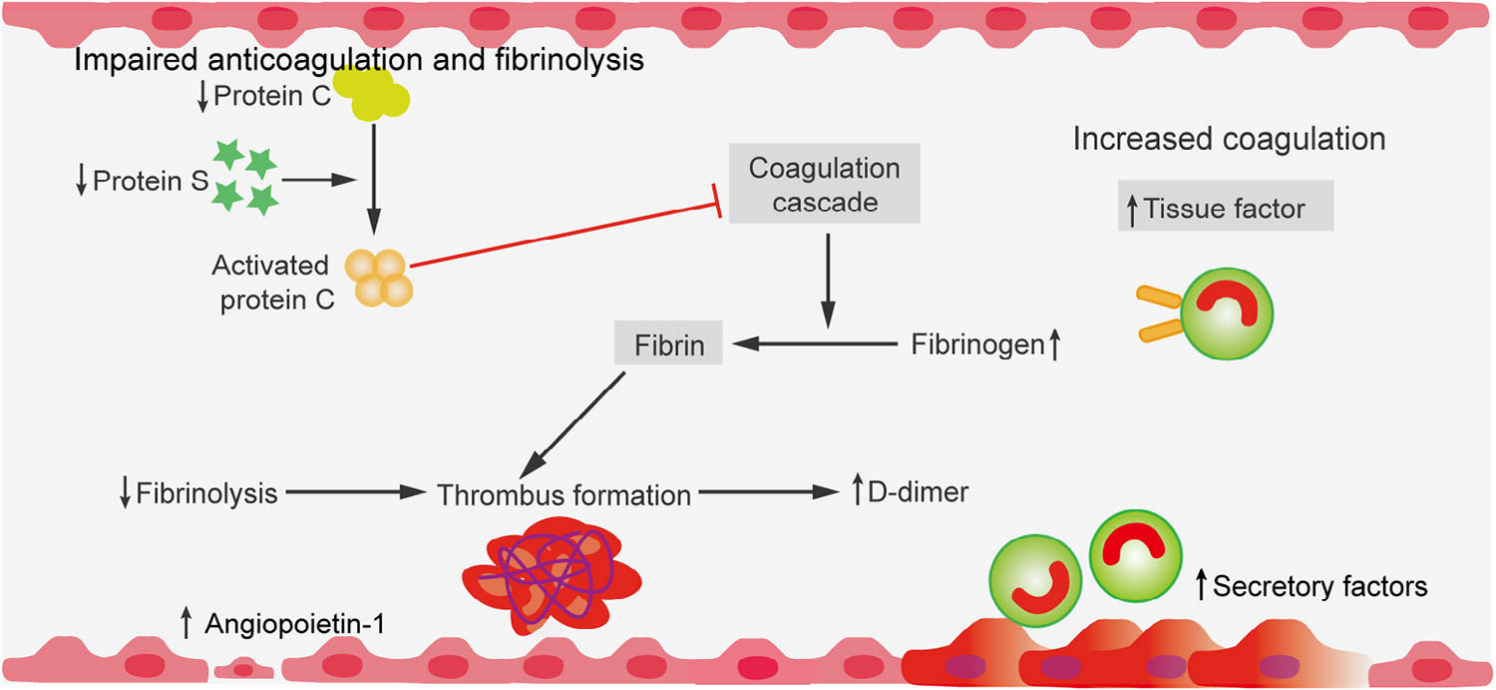
Coagulation pathways are critical in the body's response to vascular damage. When blood vessels are injured, the exposure of tissue factor (TF) triggers the initiation of the coagulation process. This exposure leads to the formation and assembly of an exogenous teninase complex, which is integral to the generation of prothrombin. Ultimately, this results in the production of thrombin (often referred to as thrombin IIa), a key enzyme that plays a central role in the formation of fibrin clots. Thrombin not only contributes to the direct formation of fibrin but also enhances the coagulation process through feedback amplification, involving the activation of an endogenous teninase system. Furthermore, the intrinsic coagulation pathway, also known as the contact pathway, plays a significant role in stabilizing the clot through thrombase cross‐linking. This process may facilitate both the initiation and the amplification of the coagulation response, ensuring that the body effectively addresses vascular injury and promotes hemostasis. Collectively, these pathways illustrate the complex interplay of factors that contribute to the coagulation process and the restoration of vascular integrity following injury.

Blood coagulation is primed at the sites of damage to the vascular endothelial cell monolayer.^103^, ^104^ The coagulation sequence is a complex process that involves a series of transformations where zymogens are converted into their active enzyme forms. This chain of events ultimately leads to the production of thrombin (IIa), a crucial enzyme that plays a key role in hemostasis by converting soluble fibrinogen into insoluble fibrin.^65^, ^66^ As the thrombin is generated, it triggers the exposure of the subendothelial layer, which is rich in molecules such as collagen and VWF. This exposure allows these molecules to interact with plasma and circulating platelets, which subsequently adhere to the damaged site. The adhered platelets become activated and begin to aggregate, leading to the formation of a primary clot that provides initial closure of the injured vessel.^105^, ^106^ In addition to forming a physical barrier to blood loss, activated platelets release a variety of vital hemostatic components from their alpha (α) and dense granules. These components include platelet activating factor, platelet factor 4 (PF4), P‐selectin, adenosine diphosphate (ADP), and polyphosphate.^107^, ^108^, ^109^ The release of these substances has significant local cellular effects, as they stimulate the recruitment and activation of immune cells such as neutrophils and monocytes. Moreover, these released factors may enhance the accessibility of acute promyelocytic leukemia (aPL), which serves as an essential cofactor for assembling the various coagulation cofactor/enzyme protein complexes necessary for effective blood coagulation.^98^, ^110^ It is important to note that, with only a few exceptions, these enzymatic reactions predominantly take place on the phospholipid surfaces of activated endothelial cells and platelets, and they require the presence of calcium ions to proceed efficiently. The basic physiological trigger for coagulation is the transbilayer receptor TF, which is normally present in the subendothelial pool throughout the vasculature.^111^, ^112^ The coagulation process is initiated through two primary pathways: the contact (endogenous) pathway and the TF (exogenous) pathway. These pathways ultimately converge at the activation of factor X, specifically when a vessel sustains injury. The binding of factor VII (FVII), which is a zymogen, to TF instigates a significant conformational change.^19^, ^113^ This change is critical, as it leads to the hydrolytic activation of FVII into its active form, FVIIa. Once activated, TF enhances the activation of factor X by FVIIa, resulting in the formation of the initial FXa molecule. Importantly, FXa is associated with exogenous tensin cellular lipoproteins that are either released from damaged cells or presented on the surfaces of activated monocytes, endothelial cells, and certain nonvascular cells. This activation cascade additionally triggers the activation of factors IX (FIX) and VIII (FVIII), which are crucial components of the coagulation process.^114^, ^115^ The coagulation process is carefully regulated and localized to the site of vascular injury by various natural anticoagulants. These anticoagulants can be classified into three distinct categories: the antithrombins, the protein C and protein S system, and the TFPI system. The regulation of blood clot dissolution, termed fibrinolysis, is governed by the plasminogen–plasmin system.^116^, ^117^ This system is activated by PAs, which facilitate the breakdown of fibrin and modulate the process of fibrin polymerization. Importantly, the activity of this fibrinolytic system can be inhibited by PAI‐1, a protein produced by several cell types, including endothelial cells, mast cells, and basophils (Figure 2).^87^, ^93^ In addition to these coagulation and anticoagulation processes, the components of the coagulation cascade work in close collaboration with platelets, which play a vital role in hemostasis.^118^ Activated platelets contribute to the coagulation process by providing surfaces that are negatively charged phospholipids, where coagulation reactions can take place.^119^ Furthermore, these platelets release microparticles (MPs) that carry TF as well as a variety of mediators, thereby enhancing the processes of hemostasis and thrombosis.^54^, ^66^ This interplay between coagulation factors and platelets underscores the complexity and interdependence of the hemostatic response.^25^, ^120^ The activation of coagulation and fibrinolysis can be seen as a direct consequence of the acute inflammatory response. When the body experiences inflammation, cytokines—small proteins important in cell signaling—are activated, leading to changes in the endothelium, or the inner lining of blood vessels.^121^, ^122^ This altered endothelial surface becomes prothrombotic, meaning that it promotes clot formation. As a result of this cytokine activation, TF is produced, triggering the activation of the extrinsic pathway of coagulation.^123^ This pathway is crucial in the body's response to injury, as it leads to the generation of proteins that activate platelets, which play a significant role in clotting. Although the fibrinolytic system—responsible for breaking down clots, which is initiated during this inflammatory response, it is soon inhibited, disrupting the delicate balance between coagulation and fibrinolysis. This inhibition results in a considerable procoagulant state, characterized by an excess of clot formation compared to breakdown.^124^, ^125^ Eventually, this dysregulation can culminate in a serious condition known as DIC. The implications of DIC are severe, as it leads to the accumulation of fibrin and the development of microthrombi—tiny clots that can obstruct blood flow. The consequences of this cascade of events can be dire, resulting in multiorgan failure and, in the worst‐case scenarios, death. Thus, understanding this interplay between coagulation and fibrinolysis in the context of inflammation is crucial for managing serious clinical outcomes.

### 2.4 Complement‐mediated hyperinflammation

The complement system is an essential part of the innate immune system, which serves as the body's first line of defense against harmful foreign microorganisms.^126^ This system consists of a complex series of proteins that work together to identify, target, and eliminate pathogens.^127^ By facilitating immune responses, the complement system is instrumental in protecting the body from infections caused by various exogenous pathogens, thereby significantly contributing to the overall immune response and the maintenance of health.^128^ In the early pathological processes of sepsis, there is an overactivation of the complement system.^129^ Upon activation, complement fragments such as C3a and C5a are released, which further promote the production of inflammatory cytokines by activating platelets, monocytes, or endothelial cells.^130^ The interactions between the complement system and the coagulation, kinin, and fibrinolytic systems form an extremely complex network within the body, amplifying and exacerbating systemic inflammatory responses.^10^ During the middle stages of sepsis, the interplay between complement system activation, inflammatory responses, and disease severity is not fully understood.^131^ However, excessive complement activation can lead to a state of heightened inflammation in the host, thereby affecting clinical outcomes.^132^ Complement activation produces C3a and C5a, which possess potent proinflammatory activities, enhancing the chemotactic effects of leukocytes and promoting inflammatory responses.^65^ Additionally, C5a can further promote coagulation by disrupting the function of endothelial glycocalyx. Moreover, the activation of inflammasomes and gasdermin D (GSDMD) is involved in the interplay between inflammation and coagulation, further illustrating the complex connection between inflammation and the complement system.^133^ Therefore, there is a close interplay between complement system activation and inflammatory responses, and they play a significant role in the development of diseases such as myocardial infarction and sepsis.

**FIGURE 3:**
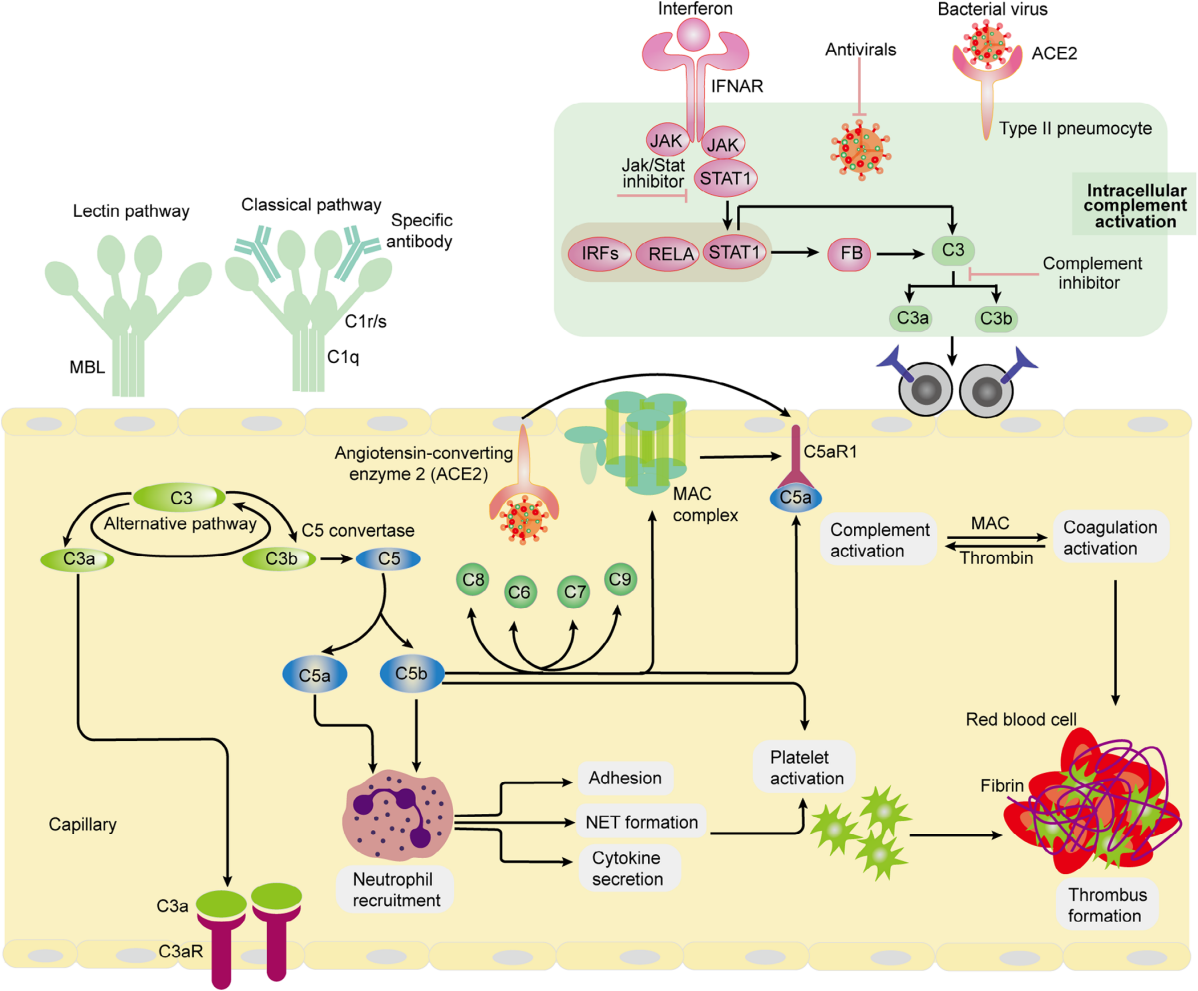
Complement activation pathways are critical biological processes that involve a series of molecular interactions leading to a robust immune response. Within this framework, inactive molecules and the resultant cleavage products are represented at the respective tails and heads of arrows, highlighting the transformation and activation of important components during the process. The intersecting lines illustrated in the pathways signify points of proteolytic cleavage, which are essential for the activation and modulation of various complement proteins. The pathways themselves are distinguished by being presented in gray italics, emphasizing their foundational role in the immune response. Furthermore, the mechanisms responsible for the detection of pathogens and damage‐associated molecular patterns (DAMPs) are prominently showcased in bold, underscoring their significance in initiating these complement activation pathways. MAC, membrane attack complex; NET, neutrophil extracellular trap.

The complement system circulating in plasma is primarily produced and secreted by the liver; however, it is important to note that complement components can also be generated by various immune and nonimmune cells.^134^ A substantial body of evidence supports the notion that hyperactivation of the complement system plays a critical role in the pathophysiological processes associated with sepsis.^135^ The mechanisms through which sepsis influences the complement system are multifaceted. Sepsis can activate the complement system directly through established pathways, including the lectin, classical, and alternative pathways (Figure 3). Additionally, sepsis can lead to endotheliopathy, characterized by endothelial cell injury and dysfunction, as well as thromboinflammation, which combines inflammation with coagulation and thrombosis.^136^ These pathological conditions can further stimulate the activation of the complement system, emphasizing a complex interplay between sepsis and immune responses.^66^ By contrast, in response to high levels of immune stimulation, complement can become uncontrolled and cause hyperinflammation.^76^ Regulation of these processes is crucial, as imbalances can have significant consequences. Under conditions of controlled inflammation, low concentrations of complement can actually inhibit the production of cytokines, leading to a situation where the concentration of C5a in serum or plasma remains extremely low.^137^ In stark contrast, when there is a high level of immune stimulation, the complement system can become dysregulated, resulting in uncontrolled complement activation and hyperinflammation.^16^ In this maladaptive state, the complement system can inflict considerable collateral damage. Neutrophils, for instance, may become unresponsive to anaphylatoxins, which severely impairs their antimicrobial capabilities (Figure 3).

Meanwhile, macrophages tend to produce excessive levels of cytokines, paving the way for cytokine storms, while endothelial cells generate TF that promotes DIC.^9^, ^138^ Furthermore, inflammation has the capacity to induce the transcription of various complement and coagulation genes, including C1r, C1s, factor B, C3, and C5, as well as fibrinogen. The presence of C5 and other products resulting from complement activation in the bloodstream, commonly observed in sepsis, signifies a loss of regulatory control over complement activation.^51^, ^53^ This deregulation gives rise to a cascade of detrimental events that compromise innate immune defenses, disrupt the balance of the clotting and fibrinolytic systems, and trigger the apoptosis of lymphocytes.^53^ This apoptotic process is believed to contribute to the profound immunosuppression frequently observed in patients suffering from sepsis. Genetic mutations affecting complement regulator genes, particularly CD55 and factor H, have been associated with an increased susceptibility to SARS‐CoV‐2 infection. This suggests that the complement system, which plays a crucial role in the immune response, may become overly active and poorly regulated in the context of sepsis. As a result, this dysregulation can contribute to a heightened inflammatory response, exacerbating the condition and potentially leading to more severe outcomes for affected individuals.

### 2.5 Complement and coagulation

**FIGURE 4:**
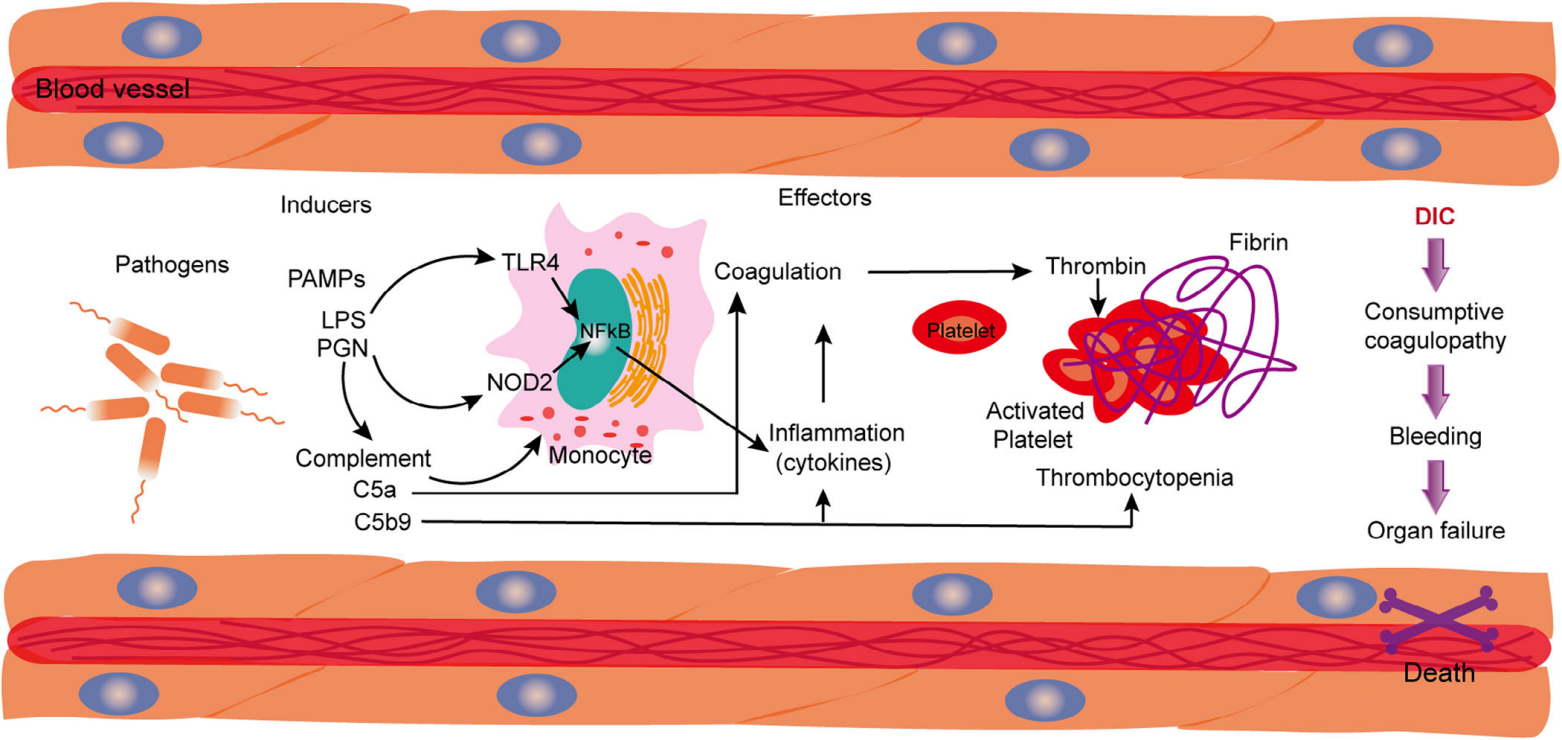
Interactions between inflammation, coagulation, and complement activation during sepsis progression. During the onset of sepsis, the inflammatory response elicited by infection can activate the coagulation system, leading to endothelial damage and the release of procoagulant substances, such as increased expression of tissue factor (TF), which activates the extrinsic coagulation pathway. The complement system is involved in excessive activation during the early pathological processes of sepsis, forming a complex network within the body through interactions with the coagulation, kinin, and fibrinolytic systems, amplifying and exacerbating the systemic inflammatory response. The complement activation products C3a and C5a possess potent proinflammatory activity, capable of activating platelets, monocytes, or endothelial cells, further promoting the production of inflammatory cytokines. Concurrently, the activation of the coagulation system can also trigger the activation of the complement system, creating a positive feedback loop. Endothelial cells are injured in sepsis, leading to a decline in the vascular endothelial barrier function, which promotes the activation of coagulation factors and the formation of thrombi. Platelets are activated in sepsis, participate in the coagulation process, and form complexes with neutrophils, promoting inflammatory responses, and thrombosis. Given the significant role of the complement system in sepsis‐associated coagulopathy, complement inhibitors may emerge as a novel strategy for treating sepsis‐associated coagulation disorders. DIC, disseminated intravascular coagulation; LPS, lipopolysaccharide; NFkB, nuclear factor kappa B; PAMPs, pathogen‐associated molecular patterns; PGN, peptidoglycan; TLR, toll‐like receptor.

The hyperactivation of both the complement and coagulation systems has been increasingly recognized as a critical aspect of the clinical syndrome associated with sepsis.^6^, ^46^ This review aims to explore the intricate mechanisms of complement activation and coagulation response triggered by the severe acute sepsis‐causing viruses. Additionally, it focuses on understanding the established connections between these immune responses, hyperinflammation, and thrombosis, which are common complications in septic patients.^3^ Recent clinical findings and emerging evidence from clinical trials suggest that targeting the complement and coagulation pathways may offer promising therapeutic benefits in the management of sepsis.^50^ Upon activation, neutrophils release a wide array of bioactive molecules, such as neutrophil elastase, cathepsin G, myeloperoxidase, and reactive oxygen species.^7^ These substances may modulate the production of coagulation factors and complement proteins, leading to complex cellular interactions that can either enhance or inhibit these pathways.^7^ One significant aspect of neutrophil activity is the release of neutrophil extracellular traps (NETs), composed of DNA and proteins that form a scaffold facilitating interactions with activated platelets (Figure 4). This mechanism creates an environment conducive to the localization of TF activity, potentially by trapping extracellular vesicles that further influence coagulation processes.^51^ Both coagulation and complement pathways can be amplified through direct interactions with NET structures, which serve as a platform for the activation of the contact and alternative pathways, respectively.^102^ Such multistep crosstalk between these systems contributes to the increased production of key components such as thrombin, C3a, and C5a, all of which have significant biological implications in the inflammatory response during sepsis. Furthermore, histones H3 and H4, which are prominent proteins found within NETs, play a crucial role in enhancing coagulation and complement activation.^15^, ^66^ They do this by inhibiting regulatory proteins like antithrombin and thrombomodulin, thereby stabilizing clots and impairing fibrinolysis initiated by tissue plasminogen activator (tPA). The surface of activated platelets is generally understood to promote coagulation due to its provision of anionic phospholipids.^50^, ^68^ However, the presence of proteins such as P‐selectin and properdin on platelet surfaces also contributes to the stabilization of C3 convertase complexes through their interactions with C3b and the water‐soluble form of C_3_ (C_3_(H_2_O)). Additionally, platelets may directly participate in complement activation by associating with C_3_(H_2_O), thereby further integrating the coagulation and complement systems in the context of sepsis.

Coagulation and complement are distinct biological systems, each playing a unique role in pathophysiological processes.^60^, ^67^ However, they exhibit several functional similarities that are often overlooked, highlighting a fascinating interplay between the two systems.^3^, ^64^ First, both systems act as essential innate defenses against external threats, such as microbial invasions. This innate defense mechanism is crucial for maintaining the body's integrity against various pathogens. Second, both coagulation and complement pathways require exogenous stimuli and changes in cell surface receptors for their activation. This criterion is vital, as it ensures that the initiation of these pathways is not only accurate but also tightly coordinated. For instance, when these systems are in proximity to the vascular endothelium, there is a reduction in the kinetic requirements for the reactions to take place. This proximity leads to a localized increase in the concentration of the necessary triggers, facilitating a more efficient response. Moreover, the reactions within both systems are organized into three distinct phases: initiation, amplification, and propagationg.^3^ This structured cascade is not merely coincidental but often operates in a self‐reinforcing manner (Figure 4). Such organization allows for multiple activation points and the capacity for both amplification and regulation—either positively or negatively. Additionally, the interactions between these cascades and other systems help to fine‐tune the body's response to various stimuli. Another critical aspect is the presence of regulatory molecules, including natural inhibitors or cofactors, that are found within the same environments as the coagulation and complement systems.^139^ These molecules play an essential role in the physiological regulation of both systems, providing a mechanism for their restraint at two levels: either through the inhibition of enzymatic activities or by limiting the binding capacity of components within the cascade. Finally, certain elements from each cascade are capable of interacting with receptors on the cell surface, which mediates various downstream biological effects.^39^ The shared characteristics of the coagulation and complement systems facilitate numerous instances of crosstalk between them, leading to their involvement in a range of clinical inflammatory and thrombotic conditions. This crosstalk emphasizes the importance of understanding how these systems interact within the broader context of human health and disease.

The complement and coagulation pathways play critical and interconnected roles in the context of sepsis.^50^ The byproducts of complement activation have the potential to enhance the clot‐forming ability of blood.^140^ This occurs through the simultaneous promotion of procoagulant proteins, which facilitate clot formation, and antifibrinolytic proteins, which deter the breakdown of clots. Concurrently, this process involves the suppression of natural anticoagulants that typically help regulate clot formation.^60^ Notably, the terminal complement complex C5b‐9 has the ability to activate TF, while the C5b‐7 initiation complex can also trigger the expression of TF through mechanisms that rely on protein disulfide isomerase.^66^ Furthermore, C5b‐9 plays a critical role in exposing phosphatidylserine on the surface of platelets, which in turn creates a conducive environment for the assembly of prothrombinase, a key component in the clotting process.^141^ Recent research has demonstrated that peptidoglycan (PGN) can elicit complement activation via the classical pathway.^136^ This activation leads to the deposition of C5b‐9 on the surface of platelets, which promotes platelet aggregation and the exposure of procoagulant activity characterized by a high concentration of phosphatidylserine.^142^ The underlying mechanism involves PGN–anti‐PGN immune complexes that facilitate the consumption of complement components. In experiments involving baboons challenged with *Escherichia coli*, the inhibition of the complement pathway using compstatin (COM), a selective C3 convertase inhibitor, was found to significantly reduce both thrombocytopenia and microvascular thrombosis.^54^ Additionally, this treatment helped to preserve the endothelial properties that prevent clotting, improve the barrier function of blood vessels, and mitigate organ injury. These findings underscore the intricate relationship between complement activation and coagulation, which together contribute to the deterioration observed in severe sepsis.^8^ Therefore, targeting the adverse effects of complement activation products, particularly during the critical phase of organ failure associated with sepsis, emerges as a promising therapeutic approach. The interaction between the coagulation and complement systems is bidirectional; complement proteins can activate the coagulation cascade, while certain enzymes involved in coagulation—such as thrombin and factor Xa—can independently activate elements within the complement cascade.^89^, ^143^ Additionally, there exists a pathway involving the anticoagulant thrombomodulin‐protein C that serves to inhibit the activation of the complement system, further illustrating the complex and reciprocal nature of these two critical biological processes.^90^ Moreover, there are various shared elements between the complement and coagulation pathways, enhancing the understanding of their interdependent roles in maintaining hemostasis and pathogen responses during sepsis. Factor XIIa plays a significant role in two critical processes within the body: the contact activation of the coagulation cascade and the activation of the complement system through the classical pathway.^144^ Its involvement in coagulation is essential for the proper formation of blood clots, while its role in the complement activation is crucial for the immune response. Furthermore, the C1 inhibitor serves as a powerful neutralizing agent. It effectively inhibits both factor XIa, which is a key player in the coagulation pathway, and the C1 component of the classical complement pathways.^99^ This dual action of the C1 inhibitor highlights its importance in regulating these interconnected systems, ensuring a balanced response to physiological challenges.

The complement and coagulation systems play a crucial role in the genesis, progression, and exacerbation of sepsis, with intricate interplay between them.^46^ Initially, the activation of the complement system is a key component of the early pathological process in sepsis.^6^ The production of complement components such as C3a and C5a possesses potent proinflammatory activity, capable of promoting the activation of leukocytes, endothelial cells, and platelets, leading to the onset of inflammatory responses.^63^ The activation of the complement system can exacerbate the coagulation dysfunction caused by sepsis through its interaction with the coagulation system, potentially leading to DIC.^18^ The activation of the coagulation system can be considered part of the initial immune response to invading pathogens, aiding in the triggering of innate defense mechanisms.^32^ However, in sepsis, the activation of the coagulation system is imbalanced, leading to a propensity for thrombosis within the microvasculature, which can progress to DIC in severe cases.^20^ TF, as the primary initiator of coagulation, increases its expression in sepsis, activating the coagulation process by forming a complex with coagulation factor FVIIa.^76^ Additionally, NETs released by neutrophils in sepsis can promote platelet adhesion, activation, and aggregation, forming thrombi, which further illustrates the interplay between the complement and coagulation systems in sepsis.^145^, ^146^ The complement system interacts with the coagulation system; while complement activation can induce inflammatory responses, excessive activation can induce dysregulation of inflammation, exacerbating inflammatory injury and worsening the condition of sepsis.^147^, ^148^ Levels of complement C3 and C4 may decrease in patients with sepsis, and their reduction is associated with the development of acute kidney injury (AKI) in sepsis.^149^ It is noteworthy that if the coagulation dysfunction in sepsis is persistent and uncorrected, it often evolves into DIC, which is closely related to the occurrence of multiple organ failure and increased mortality rates. Therefore, interventions targeting the complement and coagulation systems may offer new avenues for the treatment of sepsis‐associated coagulopathy. In summary, the interplay between the complement and coagulation systems significantly impacts the severity of the disease and patient prognosis, and inhibiting complement activation may help control coagulopathy associated with sepsis, improving patient outcomes.

## 3 THE PARTICIPANTS WITH COAGULATION AND COMPLEMENT

### 3.1 Tissue factor

TF, also referred to as coagulation factor 3 (F3), plays a crucial role in the process of blood coagulation.^66^ Its involvement in the pathological mechanisms of sepsis has been substantiated by preclinical research focused on bacterial sepsis.^150^, ^151^ Recent investigations have illuminated the role of TF in modulating pyroptosis of macrophages associated with caspase‐11, which is activated via type I interferon (IFN).^128^, ^129^ During this pyroptotic process, macrophages can release TF through extracellular vesicles that are expelled from pores formed during cell lysis.^26^ This release contributes to a rapid activation of pathological coagulation, which can exacerbate the condition.^102^, ^149^ Further insights into this area of study have revealed that certain compounds, such as dimethyl fumarate (DMF) and 4‐octyl itaconate (4‐OI), are capable of inhibiting TF‐dependent thrombin generation.^22^ This inhibition occurs through the blockade of type I IFN signaling and caspase‐11‐mediated macrophage pyroptosis, thereby preventing the subsequent release of TF in the setting of lipopolysaccharide (LPS)‐induced sepsis.^147^

Additionally, the activation of inflammasomes is critical, as they not only facilitate the processing and release of IL‐1 family cytokines but also induce the secretion of TF from activated macrophages and monocytes.^101^ The interplay between inflammasomes and innate immunity contributes to coagulation processes that can worsen the state of sepsis.^152^ Moreover, the propensity for thrombus formation during sepsis can be traced back to the simultaneous dysfunction of three significant anticoagulant pathways: antithrombin, TFPI, and the protein C system.^104^ This highlights the complexity of coagulation responses in sepsis. Consequently, strategies aimed at inhibiting the release of TFs, enhancing levels of antithrombin, and activating protein systems hold promise for ameliorating the inflammatory response observed in both in vivo and in vitro sepsis models. Collectively, these findings enhance our comprehension of the intricate immune mechanisms underlying coagulopathy and pave the way for future research endeavors targeting infection‐induced sepsis in preclinical contexts.

### 3.2 Platelets

Platelets, which are small non‐nucleated cells within the bloodstream, have a critical role in sepsis.^148^ They not only contribute to the homeostasis of the coagulation system but also actively participate in immune regulation.^153^ Upon activation, platelets release proteases and bactericidal peptides.^154^ Additionally, they collaborate with white blood cells (neutrophils and monocytes) to modulate immune responses.^145^ Remarkably, scientific investigations have highlighted the significant impact of deleting the *STING* gene in platelets on platelet activity levels.^155^ This genetic manipulation leads to a decreased production of sepsis‐induced NETs and thrombosis formation, ultimately enhancing the survival rate of mice.^156^ Another intriguing study, utilizing mice lacking the *GSDMD* gene specific to platelets, demonstrated that platelet pyroptosis triggered by GSDMD results in the release of oxidized mitochondrial DNA (ox‐mtDNA), consequently promoting the formation of NETs.^146^ Succinctly, this creates a positive feedback loop that leads to an excessive release of inflammatory cytokines. However, when platelet pyroptosis was inhibited, the survival rate of mice suffering from cecal ligation and puncture (CLP) induced septicemia significantly improved.^157^ Furthermore, it has been discovered that PF4 effectively activates the clotting contact pathway by preventing the fragmentation of cfDNA and NETs into shorter fragments and single‐stranded cfDNA.^158^ As a result, the formation of cfDNA and NETs clots is hindered, thus offering valuable insights for the development of clinical interventions for sepsis.

### 3.3 GSDMD

Coagulation not only plays a vital function in the physiological hemostasis process, but also plays an important role in the body's anti‐infection immune response.^98^ GSDMD plays a pivotal role in the coagulation process during sepsis.^123^ In sepsis, the activation of GSDMD facilitates the externalization of phosphatidylserine, thereby augmenting the procoagulant activity of TF on the surface of the cell membrane. This initiates the coagulation cascade, leading to the occurrence of DIC.^133^, ^150^ The type I IFN pathway, activated during bacterial infection, induces the activation of caspase‐11 and its downstream target GSDMD, thereby precipitating the formation of DIC.^19^, ^50^, ^93^ The activation of GSDMD can indirectly affect the complement system by modulating the inflammatory environment.^159^ The release of IL‐1β and other cytokines can amplify the complement activation cascade, leading to a more robust inflammatory response.^160^ The interaction between GSDMD and the complement system offers potential therapeutic targets for the treatment of sepsis. Modulating the activity of GSDMD or the complement system could help control the inflammatory response and reduce the severity of sepsis.^161^ Consequently, interventions targeting the functionality of GSDMD offer a potential opportunity for the prevention and treatment of sepsis‐associated DIC, rendering it an important therapeutic target for pharmacological intervention in the management of sepsis.

### 3.4 HMGB1

Clinical studies have shown that the serum high mobility group protein‐1 (HMGB1) level of normal people is very low, while the expression level of HMGB1 in the serum of patients with sepsis and hemorrhagic shock is significantly increased, and the level of HMGB1 in the death group is higher than that in the survival group.^162^ In sepsis, the release of HMGB1 into the bloodstream by immune cells mediates the activation of caspase‐11 and GSDMD, leading to a hypercoagulable state, the induction of DIC, and resulting in multiple organ failure.^143^, ^163^ HMGB1 is a typical risk warning molecule. Previous studies have found that platelets can release a large amount of endogenous HMGB1 during the development of sepsis, which further leads to platelet aggregation and activation and plays a protective role in the body.^164^, ^165^, ^166^ Furthermore, HMGB1 facilitates the formation of NETs, which are capable of capturing bacteria and activating platelets, thus promoting thrombus formation.^163^, ^167^ Given HMGB1's role in the coagulopathy associated with sepsis, it may serve as a potential therapeutic target for anticoagulant treatment, with interventions aimed at HMGB1 potentially improving the coagulation function in septic patients.

In sepsis, the effect of HMGB1 in the complement system is particularly relevant due to its ability to exacerbate the inflammatory response and contribute to the development of a cytokine storm, a hallmark of severe sepsis and septic shock.^164^, ^168^, ^169^, ^170^ HMGB1 is an important factor mediating infection, tissue injury, and inflammation. Normally, HMGB1 is primarily located in the nucleus and acts as a chaperone for DNA to bind to chromatin, but it can also shuttle from the nucleus to the cytoplasm under various stress conditions.^143^, ^171^ HMGB1 is actively secreted by macrophages and other cells or after infection and release from damaged/necrotic cells.^163^, ^172^, ^173^ Its function is to recruit inflammatory cells and mediate signaling among macrophages, dendritic cells, and natural killer cells. Extracellular HMGB1 can activate endothelial cells, promote angiogenesis, enhance hematopoietic stem cell migration, and trigger local or systemic inflammation.^174^, ^175^ Given these multifaceted roles, HMGB1 represents an important therapeutic target for interventions aimed at modulating the complement system and treating sepsis. Strategies to inhibit HMGB1, such as the use of neutralizing antibodies or small molecules that interfere with its release or activity, could potentially improve outcomes in patients with sepsis by reducing the hypercoagulable state and associated organ damage.

### 3.5 STING1

T platelets play a pivotal role in the abnormal coagulation function in sepsis. Beyond their traditional involvement in the coagulation process, platelets also possess immune regulatory functions.^39^, ^176^ Platelets play a pivotal role in the abnormal coagulation function in sepsis. Beyond their traditional involvement in the coagulation process, platelets also possess immune regulatory functions.^54^, ^89^, ^144^, ^177^ This release is essential for platelet activation and highly dependent on the assembly of the SNARE complex.^90^, ^152^ STING interacts with the secretory protein STXBP2 to maintain the effective assembly of the SNARE complex, while the STING agonist cGAMP can enhance this binding, further promoting the secretion and functional activity of platelet granules.^16^, ^104^, ^178^ In addition, research has identified potential binding sites between STING and STXBP2,^179^, ^180^, ^181^ and the design of corresponding peptides can inhibit their binding, thereby suppressing the level of platelet activity and the formation capacity of NETs in vitro,^182^, ^183^ as well as alleviating the formation of septic thrombi.^184^ The absence of platelet STING has been found to alleviate the formation of microthrombi and NETs structures in the livers and lungs of septic mice induced by sepsis, reduce liver and lung damage and coagulation abnormality‐related indicators, and enhance the survival rate of mice.^185^ These findings reveal the mechanism by which platelet STING regulates thrombus formation in sepsis infection and provide potential intervention strategies for the treatment of sepsis.

Research has demonstrated that myeloid STING plays a crucial role in modulating coagulation during bacterial infections, functioning through a mechanism that does not rely on the type I IFN response.^185^ In terms of its mechanism of action, STING interacts with ITPR1, which regulates the release of calcium from the endoplasmic reticulum (ER) within macrophages and monocytes.^186^, ^187^ The increase of cytosolic Ca^2+^ that is dependent on STING subsequently promotes the cleavage and activation of GSDMD.^139^, ^180^, ^182^ This activation is significant as it initiates the release of TF F3, which is a key promoter of coagulation processes. Moreover, the pathways associated with STING have profound implications on systemic coagulation. Specifically, either genetic alterations or pharmacological interventions that inhibit the STING‐GSDMD‐F3 pathway can impede systemic coagulation, leading to improved survival outcomes in animal models of sepsis. This suggests that targeting this pathway may provide a therapeutic strategy in critically ill patients.^188^, ^189^ Furthermore, clinical observations indicate that the upregulation of the STING pathway correlates with increased severity and mortality rates associated with DIC in septic patients, highlighting its potential as a biomarker for disease prognosis.^106^ In short, STING‐mediated blood coagulation activation, the blood coagulation activation depends on the ER calcium release, and independent of the type I IFN response, inhibit lethal infection of the blood coagulation activation. Direct targets for STING, ATP2A2, it relies on ER calcium intake limit STING‐mediated F3 release, and STING‐mediated GSDMD cracking decided to release and death F3 clotting.^181^, ^190^, ^191^ Finally, STING‐GSDMD pathways associated with sepsis patients with DIC severity. Thus, STING is a key regulator of coagulation during lethal bacterial infections. Platelet STING thrombosis in the regulation of sepsis infection play a major role, and further reveal the pathogenesis of sepsis, offers the potential for sepsis treatment intervention strategy.

### 3.6 C3/C5

Sepsis is a condition that arises from a dysregulated immune response to infection, where the body's defenses become impaired. The complement system is a vital component of the host's defense mechanism against various pathogens, operating through three main pathways: the classical, lectin, and alternative pathways.^10^, ^68^ Each of these pathways plays a crucial role in activating the complement system, leading to the proteolytic cleavage of key proteins such as C3 and C5.^8^, ^50^, ^66^ This process ultimately results in the formation of terminal complement complexes, which are necessary for combating infections. However, it is important to note that when there is excessive activation of the complement system, it can trigger a hyperinflammatory response in the host, which may exacerbate the condition.^109^, ^192^ The complement system serves as an essential part of the innate immune system, contributing significantly to immune regulation and the maintenance of immune homeostasis. Within this complex system, complement component C3 stands out as a central element, as it is crucial for both the classical and alternative pathways of complement activation.^110^ The activities of C3 highlight its importance in effectively managing and orchestrating the immune response to ensure that the body can adequately respond to infections while avoiding an overactive inflammatory state that could be detrimental to the host.^48^ The chemical component is glycoprotein, composed of two peptide chains, α and β, connected by disulfide bonds. It is the highest content of all complement components in serum. C3 convertase ACTS on the C3 cracking of C3a and C3b.^9^, ^65^ C3a, which is released into body fluids, has anaphylatoxin and chemotactic activity. C3b combines with C4b2a to become C4b2a3b, the C5 convertase in the classical activation pathway, and C5 is the substrate of this enzyme. C3b also has adhesion and regulate immune phagocytosis.^11^, ^12^ C3 defects of the body prone to suppurative infection, closely associated with many diseases, especially the blood coagulation disorder caused by sepsis.

In the early stages of sepsis, the activation of the complement system triggers the release of potent proinflammatory mediators, specifically C3a and C5a. These molecules play critical roles in enhancing the immune response by facilitating the recruitment and activation of various immune cells, including leukocytes, as well as endothelial cells and platelets.^16^, ^17^ While the activation of the complement system is a vital element of the body's initial defense mechanisms, it can become detrimental if left unregulated. Excessive or uncontrolled complement activation can result in tissue damage, potentially leading to severe complications such as organ failure.^51^, ^76^ Furthermore, sepsis is characterized by a dysregulation in the activation of the coagulation system, which creates a tendency for thrombosis within the microvasculature. One of the most severe consequences of this sepsis‐induced coagulopathy (SIC) is DIC. This condition is marked not only by thrombosis but can also involve bleeding, attributed to the depletion of coagulation factors, anticoagulant proteins, and platelets during the coagulation process.^193^ A major contributor to the initiation of coagulation is TF, which promotes coagulation by activating coagulation factors FX and FIX in conjunction with the factor FVIIa. TF is predominantly expressed by perivascular cells, including fibroblasts, pericytes, and epithelial cells, highlighting its crucial role in maintaining hemostasis and vascular integrity.^52^, ^193^ In response to various microbial agents and a range of inflammatory stimuli, including cytokines and complement components, TF can be expressed on endothelial cells, monocytes, and macrophages. Under pathological conditions, an abundance of biologically active TFs is found within macrovesicles derived from multiple cell types.^53^, ^194^ These macrovesicles can interact with other cells, such as activated platelets, neutrophils, and endothelial cells, thereby strengthening the coagulation cascade. In addition to their role in coagulation, TF, and various coagulation factors such as FVIIa, FXa, thrombin, and fibrin can generate proinflammatory signals by activating members of the G‐protein‐coupled protease‐activated receptor family.^9^, ^66^ Additionally, the relationship between complement factors and thromboplastin is reciprocal, as each can activate the other, further contributing to the complex interplay of hemostasis and inflammation in sepsis. For instance, the proteins FIXa, FXa, FXIa, and thrombin are capable of converting complement components C3 and C5 into their active peptides, C3a and C5a, respectively.^17^ This process underscores the intricate relationship between the complement system and the coagulation cascade. Notably, C5a, alongside the MAC (C5b‐9), has a significant role in upregulating TF expression in endothelial cells.^112^ This expression is crucial as it contributes to the procoagulant state observed during inflammatory responses. Moreover, C5a can exacerbate coagulation by disrupting the function of the endothelial glycocalyx, which serves as a protective barrier for the endothelium. In summation, complement proteins C3 and C5 are pivotal in the onset and progression of sepsis, highlighting the critical connection that inflammasome activation represents between the complement system and coagulation pathways.

## 4 INTERVENTION THERAPIES OF COAGULATION AND COMPLEMENT

As a result of the considerable adverse effect of sepsis on families in relation to health, emotions, and economy, scientists and medical professionals have devoted their efforts to investigating sepsis with the aim of developing efficacious medications for its management. In recent years, crucial strides have been made in our comprehension of the pathogenesis and progression of sepsis. Despite the absence of clinical drugs for treating sepsis, numerous inhibitors with potential therapeutic value have been developed both in vivo and in vitro, thanks to the relentless efforts of researchers (Table 1). Some of these inhibitors have been granted approval for further assessment in preclinical investigations (Table 2). This valuable information serves as a guide for the development of sepsis drugs and provides a solid research foundation for the clinical treatment of sepsis.

**TABLE 1:** Selected inhibitors for putatively targeting immunocoagulation pathways on sepsis in preclinical studies.

Drug	Year	Target	Mechanism	Usage	Model	Refs.
Ac‐FLTD‐CMK	2018	CASP1/4/5/11	Inhibit the generation of GSDMD‐N by blocking CASP 1/4/5/11	In vitro: 10 µM In vivo: N/A	In vitro: BMDM In vivo: N/A	^195^
Z‐IETD‐FMK	2018	CASP8	Pyroptosis is inhibited by blocking the production of GSDMD‐N mediated by CASP8 activity	In vitro: 10 µM In vivo: N/A	In vitro: BMDM In vivo: N/A	^117^
U73122	2018	PLCG1	Inhibits the pyroptosis by blocking the generation of GSDMD‐N	In vitro: 10 µM In vivo: 30 mg/kg	In vitro: BMDM In vivo: CLP (mouse)	^117^, ^196^
H‐151	2018, 2020	STING1	Transmembrane cysteine residues are covalently bound to STING to inhibit STING1	In vitro: 2 µM In vivo: 750 nM/mice	In vitro: primary human or mouse macrophages In vivo: Trex1−/− mice	^179^, ^197^
YQ128	2019	NLRP3	Selectively inhibits NLRP3 inflammasome and has blood–brain barrier permeability	In vitro: 10–100 µM In vivo: 10–20 mg/kg	In vitro: BMDM, J774A.1 In vivo: endotoxemia (mouse)	^198^
FPS‐ZM1	2019	AGER	AGER V‐domain‐mediated high‐affinity inhibitors for ligand binding	In vitro: 0.1–1 µM In vivo: 10 mg/kg, 75 µg/day	In vitro: BMDM In vivo: endotoxemia, *Acinetobacter baumannii* infection (mouse)	^130^, ^199^
Zileuton	2019	ALOX5	Inhibits lipid peroxidation by blocking ALOX5	In vitro: 5 µM In vivo: 30 mg/kg	In vitro: BMDM In vivo: endotoxemia (mouse)	^199^
Disulfiram	2020	GSDMD	Pyroptosis is inhibited by preventing the formation of GSDMD pores	In vitro: 1–30 µM In vivo: 15–50 mg/kg	In vitro: BMDM, THP1 In vivo: endotoxemia (mouse)	^200^
TUDCA	2020	ER stress and Ca^2+^	Inhibits ER stress and calcium release	In vitro: 50 µM In vivo: 200 mg/kg	In vitro: THP1 In vivo: CLP (mouse)	^117^
4PBA	2020	ER stress and Ca^2+^	Inhibits ER stress and calcium release	In vitro: 1 mM In vivo: 20 mg/kg	In vitro: THP1 In vivo: CLP (mouse)	^117^
DMF	2023	Type I IFN and TF	block type I IFN‐ and caspase‐11‐mediated macrophage pyroptosis, and subsequent TF release	In vitro: 10 µM In vivo: 50 mg/kg	In vitro: BMDMs In vivo: LPS (mouse)	^26^
4‐OI	2023	Type I IFN and TF	block type I IFN‐ and caspase‐11‐mediated macrophage pyroptosis, and subsequent TF release	In vitro: 250 µM In vivo: 50 mg/kg	In vitro: BMDMs In vivo: LPS (mouse)	^26^

Abbreviations: BMDM, bone marrow derived macrophages; AGER, advanced glycation end‐product receptor; 4‐OI, 4‐octyl itaconate; 4PBA, 4‐phenyl butyric acid; Ac‐FLTD‐CMK, N‐acetyl‐Phe‐Leu‐Thr‐Asp‐chloromethylketone; DMF, dimethyl fumarate; ER, endoplasmic reticulum; GSDMD, gasdermin D; IFN, interferon; LPS, lipopolysaccharide; TF, tissue factor; TUDCA, tauroursodeoxycholic acid.

**TABLE 2:** Current clinical studies that aim coagulation and complement in sepsis.

Treatment	Mechanism	Study phase	Clinicaltrials.gov identifier	Primary outcome	Source
Ulinastatin	A broad‐spectrum protease inhibitor	Phase 3	NCT05391789	Sequential organ failure asses (SOFA) of day 5, compared with the baseline	–
Antithrombin alfa	–	Phase 2	NCT00506519	Patients alive on day 28 having had an improvement in DIC score and having had no worsening on organ failure score	–
Nafamostat mesilate	–	Phase 4	NCT06078839	All‐cause mortality in ICU (time frame: 7 days)	–
Heparin sodium	Heparin inhibits caspase‐11 activation by blocking cytosolic delivery of LPS	Phase 3	NCT04861922	Death from all causes at 28 days	^123^, ^201^, ^202^, ^203^
Activated protein C and corticosteroids	–	Phase 3	NCT00625209	90‐day mortality	^204^, ^205^
Nafamostat mesilate	–	Phase 4	NCT06078839	All‐cause mortality in ICU (time frame: 7 days)	–
Dociparstat sodium	Targeted inhibition of HMGB1 protein	Phase 3	–	–	–
SB‐17170	Targeted inhibition of HMGB1 protein	Phase 1	–	–	–
Ravulizumab (ultomiris)	Targeted inhibition of C5	Phase 3	NCT03748823	Ctrough serum concentration of ravulizumab (time frame: predose at day 71)	^206^
Pegcetacoplan (EmpaveliTM)	Targeted inhibition of C3	Phase 2	NCT04901936	Pegcetacoplan serum concentrations over the course of the 16‐week treatment period	^207^
Avacopan (Taveos)	Targeted inhibition of C5a	Phase 4	NCT06072482	Percentage of participants experiencing treatment‐emergent adverse events (TEAEs; time frame: up to month 60)	^208^

Abbreviations: DIC, disseminated intravascular coagulation; HMGB1, high mobility group protein‐1; ICU, intensive care unit; LPS, lipopolysaccharide.

## 5 CONCLUSIONS AND PROSPECTS

There are several primary approaches for immunotherapy of sepsis in current research and clinical practice: Cytokine therapy, some cytokines such as granulocyte‐macrophage colony‐stimulating factor (GM‐CSF), IFN‐γ, IL‐7, and IL‐15, can modulate immune cell functions and assist in reversing the immunosuppressive state in septic patients. Immune checkpoint inhibitors, such as PD‐1/PD‐L1 antibody, anti‐PD‐1/PD‐L1 therapy improves survival in a mouse model of sepsis. Thymosin α1, as an immunomodulator, thymosin α1 may contribute to enhancing the immune function of septic patients. Intravenous immunoglobulin (IVIG), which contains a variety of antibodies, can effectively neutralize the endotoxin and exotoxin released by the bacteria and regulate the immune response. Although controversial in sepsis treatment, some studies suggest that it may help to improve clinical outcomes. Treatment with mesenchymal stem cells (MSCs), which have tissue repair and immunomodulatory functions, has demonstrated potential to reduce inflammation and improve lung injury in septic animal models. The regulation of myeloid‐derived suppressor cells (MDSCs), which play an immunosuppressive role in sepsis, may help to restore normal immune function by regulating MDSCs. The regulation of anti‐inflammatory/proinflammatory balance through the use of drugs or other therapeutic means to regulate the inflammatory response in patients with sepsis may help to reduce immunosuppression and improve prognosis. Personalized immunotherapy, tailored immunotherapeutic strategies based on the immune status of septic patients, such as using inflammatory inhibitors or immune activators depending on serum ferritin levels or monocyte human leukocyte antigen‐DR (HLA‐DR) levels. Among these strategies, some have entered clinical trial phases, while others are still in the laboratory research stage. However, progress in the immunotherapy of coagulation and complement systems for sepsis treatment has been slow, and more research is needed to determine the optimal treatment plans and timing.

In the realm of sepsis research, the interplay between the coagulation and complement systems stands as a pivotal avenue of investigation. Future foundational studies are imperative to elucidate the modulation of coagulation factors and anticoagulant proteins to govern the activation and suppression of the coagulation system. The overactivation of the complement system in sepsis can precipitate severe inflammatory responses and organ damage. Studies have indicated a significant complement activation in sepsis patients, yet the correlation with clinical outcomes remains ambiguous, underscoring the necessity for further research into the specific mechanisms of the complement system's role in sepsis. Moreover, the identification and validation of biomarkers for early diagnosis and prognostic assessment of sepsis constitute a focal point of current research. For instance, IL‐6, complement C1q, and the SIC score are deemed to possess predictive value for early warning and prognosis in sepsis, and future research is warranted to further validate the clinical application value of these biomarkers. The coagulation system and the complement system are intricately interrelated, with this interplay being particularly pronounced in sepsis. Future research endeavors must delve into the specific mechanisms of this interaction and explore how to modulate it for the treatment of sepsis. For example, research suggests that complement activation and the activation of the coagulation system are interlinked in sepsis, but the precise mechanisms require further investigation. Sepsis research necessitates interdisciplinary collaboration, encompassing basic medical science, clinical medicine, immunology, and molecular biology. Future research must integrate knowledge from these fields to comprehensively understand the pathophysiological mechanisms of sepsis and to develop more effective therapeutic strategies. Ultimately, high‐quality clinical trials are pivotal in validating the efficacy of new treatment strategies. Future research must design and implement more randomized controlled trials to assess the efficacy of new drugs and treatment methods in the management of sepsis. For example, studies indicate that early administration of norepinephrine can enhance the survival rate of patients with septic shock, yet additional clinical trials are imperative to confirm its effectiveness. Through these research directions, future studies can provide more scientific evidence and novel therapeutic approaches for the treatment of sepsis.

This review focuses on the aggregation of molecular mechanisms and potential therapeutic targets related to coagulation and complement that occur during sepsis, aiming to understand the process of immune response dysregulation of coagulation and complement in the development of sepsis. The views proposed in this paper are based on the molecular regulatory mechanisms of sepsis‐related coagulation and complement cross‐linking described in previous studies, and on this basis, the potential regulatory targets are proposed, so as to provide more reference for the subsequent treatment of sepsis. This article also discusses in detail the discovery of the main intervention therapy (clinical and basic) drugs in the process of sepsis and the subsequent clinical research progress. Finally, the main targets and therapeutic interventions of activated host responses in the coagulation and complement systems are proposed to better understand the effective interventions that can be taken during the developmental stage of sepsis, aiming to interpret the multiorgan failure caused by the disorder of complement and coagulation triggered response that is characteristic of sepsis. This review provides the clinical potential treatment of sepsis more valuable reference.

## AUTHOR CONTRIBUTIONS

Honghong Jiang and Wendan Zhang conceived, wrote, and edited the manuscript. Wendan Zhang, Wei Shang, and Zhichun Feng edited and revised the manuscript. Yiming Guo, Qihang Wang, Yiran Wang, Dingchuan Peng, Yigong Fang, Lei Yan, Zhuolin Ruan, Sheng Zhang, and Yong Zhao provided significant assistance. All the authors have read and approved the final manuscript.

## CONFLICT OF INTEREST STATEMENT

The authors declare no conflicts of interest.

## ETHICS STATEMENT

Not applicable.

## Data Availability

Not applicable.
